# A simple and useful method for evaluation of oxidative stress in vivo by spectrofluorometric estimation of urinary pteridines

**DOI:** 10.1038/s41598-020-67681-4

**Published:** 2020-07-08

**Authors:** Ichiro Wakabayashi, Mamoru Nakanishi, Makoto Ohki, Akira Suehiro, Kagehiro Uchida

**Affiliations:** 1 Department of Environmental and Preventive Medicine, Hyogo College of Medicine, Mukogawa-cho 1-1, Nishinomiya, Hyogo 663-8501 Japan; 2 Mibyoumarker Laboratory Co., Ltd., Osaka, 530-0043 Japan; 3 General Education Center, Hyogo University of Health Sciences, Hyogo, 650-8530 Japan

**Keywords:** Diagnostic markers, Risk factors

## Abstract

Pteridine derivatives are intermediate metabolites of folic acid and its cofactors. Oxidized-form pteridines, but not reduced-form pteridines, are fluorescent substances. The purpose of this study was to clarify whether oxidized-form pteridine level in urine, estimated by spectrofluorometry, reflects oxidative stress in vivo. The subjects were healthy middle-aged men (n = 258). Urinary pteridine level was estimated by spectrofluorometry with an excitation wavelength of 360 nm and an emission wavelength of 450 nm. Relationships of urinary pteridines with oxidative stress markers (urinary DNA/RNA oxidation products and 15-isoprostane F_2t_) and with smoking were analyzed. Concentrations of pteridines, DNA/RNA oxidation products and 15-isoprostane F_2t_ were used after logarithmic transformation in linear analyses. Pteridine levels were significantly correlated with levels of DNA/RNA oxidation products (Pearson’s correlation coefficient: 0.626, *p* < 0.01) and 15-isoprostane F_2t_ (Pearson’s correlation coefficient: 0.695, *p* < 0.01). These correlations were not confounded by age, body mass index, history of smoking and estimated glomerular filtration rate in multivariate analysis. The mean urinary pteridine level was significantly higher in heavy smokers (16 cigarettes or more per day) than in nonsmokers and light smokers (less than 16 cigarettes per day) and was higher in light smokers than in nonsmokers. Thus, urinary fluorometric pteridine levels were shown to be associated with known biomarkers of oxidative stress as well as smoking, which causes oxidative stress in vivo. We propose spectrofluorometrical estimation of urinary pteridines as a simple and useful method for evaluation of oxidative stress in vivo.

## Introduction

Pteridines are aromatic chemical compounds that are composed of fused pyrazine and pyrimidine rings and contain a wide variety of substitutions on the heterocyclic structure. There are a variety of pteridines in intermediate metabolites of folic acid and its cofactors. Guanosine triphosphate is metabolized into tetrahydrobiopterine through dihydroneopterin, which is oxidized to neopterin. Neopterin is released from macrophages by stimulation with interferon-γ^1,2^. Therefore, urinary neopterin reflects immune activation during inflammation and has been used as a biomarker of cancer^3,4^ and cardiovascular disease^5^ as well as infectious disease^6^. Tetrahydrobiopterine is a biologically important unconjugated-type pteridine and is oxidized to other pteridines including biopterin, xanthopterin, isoxanthopterin and pterin.

There are two major methods, namely capillary electrophoresis (CE) and high-performance liquid chromatography (HPLC), for analysis of pteridines in urine^7^. The advantages of CE are reduced sample volume, direct injection and a variety of separation modes. However, applicability of CE is limited by poor reproducibility related to undesirable matrix and analyte adsorbtion on the capillary walls. Instead of the UV absorbance method, laser-induced fluorescence (CE-LIF) has been developed as a high-resolution separation technique for analysis of polar-ionic compounds. HPLC and subsequent fluorescence detection (HPLC-FD) has been developed as a method for detection of intrinsic fluorescence displayed by pteridines. HPLC-mass spectrometry (HPLC–MS) is a recent breakthrough for quantitative determination of pteridines and is suitable for analysis of polar urinary metabolites^7^. During ultraviolet radiation, folic acid is degraded to 6-formylpterin and pterin-6-carboxylic acid, which generate reactive oxygen species^8,9^. These oxidized-form pteridine derivatives in urine are fluorescent substances, while reduced-form pteridines emit little fluorescence^10^. In the present study, we applied this characteristic of pteridine derivatives for estimation of oxidative stress level in vivo.

Oxidative stress contributes to aging and the development of a variety of diseases including cancer and cardiovascular disease^11^. Pteridine derivatives have been reported to modulate oxidative stress^12^. Moreover, neopterin levels in serum, urine and ascitic fluid were suggested to be an indirect estimate of the degree of oxidative stress in patients with cancer^13^. However, it remains unknown whether and how urinary pteridine levels are affected by oxidative stress in vivo in healthy persons. We hypothesized that oxidized-form pteridines in urine reflect an increase in oxidative stress in vivo. The purpose of this study was therefore to clarify whether urinary oxidized-form pteridines, estimated by spectrofluorometry, reflect oxidative stress in healthy persons.

As known biomarkers of oxidative stress in urine, we used DNA/RNA oxidation damage products and 15-isoprostane F_2t_. The former biomarkers are produced by oxidative damage to DNA and RNA and are constituted by multiple oxidized guanine species including 8-hydroxy-2′-deoxyguanosine from DNA, 8-hydroxyguanosine from RNA, and 8-hydroxyguanine from either DNA or RNA^14^. 15-Isoprostane F_2t_ is a prostaglandin-like compound produced by free radical-mediated peroxidation of lipoprotein^15^. Smoking is known to cause an increase in oxidative stress^16,17^, which is involved in the pathogenesis of a variety of diseases including atherosclerotic vascular diseases^18,19^. Thus, we also investigated the relationship between smoking and urinary pteridines and compared this relationship with relationships between smoking and the known oxidative-stress markers such as DNA/RNA oxidation damage products and 15-isoprostane F_2t_.

## Results

### Levels of pteridine derivatives in urine

We measured concentrations of major oxidized-form pteridine derivatives in urine by HPLC. The medians with interquartile ranges in parentheses of the major derivatives (μM) were as follows: pterin-6-carboxylic acid, 0.29 (0.19, 0.40); neopterin, 1.55 (1.14, 2.01); xanthopterin, 0.21 (0.17, 0.28); isoxanthopterin, 0.35 (0.22, 0.57); biopterin, 1.63 (0.91, 2.44); and pterin, 0.92 (0.62, 1.14). Thus, the two most abundant oxidized-form pteridine derivatives in urine were neopterin and biopterin and the next-most abundant derivative was pterin.

### Excitation and emission wavelengths used in fluorescence measurement for estimation of pteridines in urine

**Figure 1:**
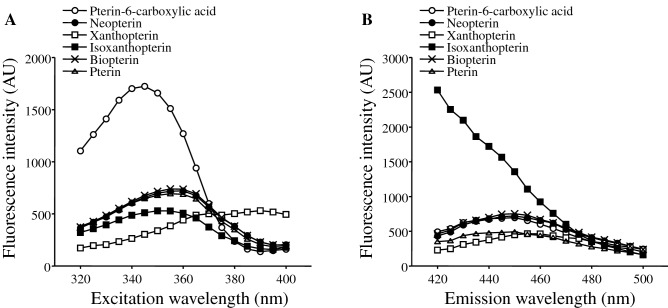
Fluorescence intensity of each pteridine derivative (1 μM) measured at different excitation wavelengths with an emission wavelength fixed at 450 nm (**A**) and at different emission wavelengths with an excitation wavelength fixed at 360 nm (**B**).

In an original study on fluorometric measurement of pteridines by Trehan and Noronha^20^, 360 mm and 450 nm were used as excitation and emission wavelengths, respectively. First, we verified these wavelengths for measurement of oxidized-form pteridines in urine. Figure 1A shows relationships of excitation wavelengths with fluorescence intensity in measurement of each pteridine derivative at an emission wavelength of 450 nm. The maximum fluorescence intensities of the above major pteridine derivatives in urine, including biopterin and neopterin, were obtained at an excitation wavelength of about 360 nm. When the excitation wavelength was fixed at 360 nm, the maximum fluorescence intensities of the major pteridine derivatives were detected at an emission wavelength of about 450 nm (Fig. 1B). We therefore determined 360 nm and 450 nm as optimum excitation and emission wavelengths to estimate levels of oxidized-form pteridines in urine.

### Distribution of urinary pteridine levels in overall subjects

**Figure 2:**
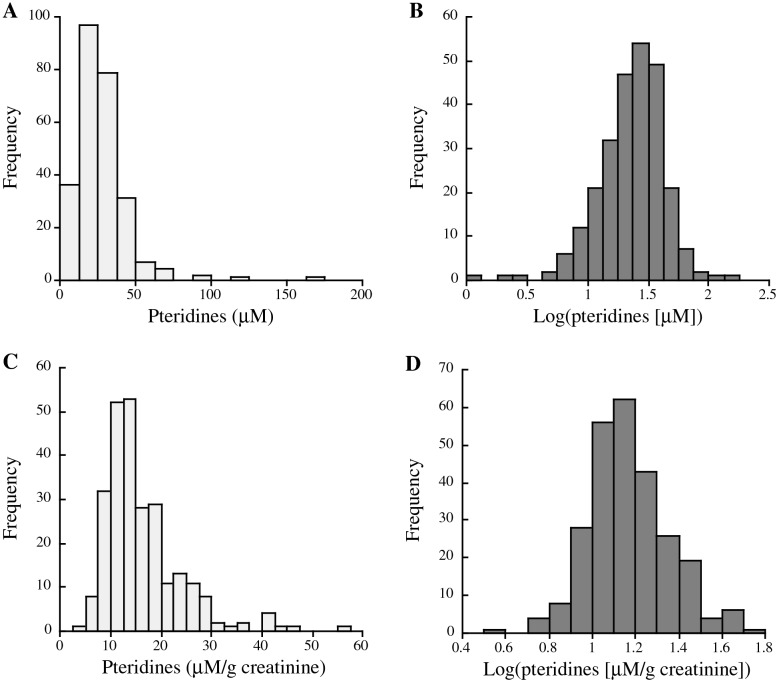
Histograms of urinary fluorometric pteridine levels before creatinine correction without (**A**) and with (**B**) logarithmic transformation and urinary fluorometric pteridine levels after creatinine correction without (**C**) and with (**D**) logarithmic transformation.

Histograms of urinary pteridine levels before and after their logarithmic transformation are shown in Fig. 2. Both urinary pteridine levels with and without creatinine correction showed a normal distribution not before but after logarithmic transformation.

### Characteristics of overall participants and comparisons of subjects in the three tertile groups for fluorometric pteridine level in urine

Table 1 shows characteristics of the overall participants and the participant groups of tertiles for urinary pteridines. The age of the participants was significantly older in the 3rd tertile for pteridines than in the 1st tertile. The percentage of smokers was significantly higher in the 3rd tertile for pteridines than in the 1st and 2nd tertiles. Among the three tertile groups for pteridines, there were no significant differences in other cardiovascular risk factors including body mass index (BMI), blood pressure, blood lipids (triglycerides, HDL cholesterol and LDL cholesterol) and fasting sugar.

**Table 1:** Characteristics of the overall subjects and subjects in the three tertile groups for urinary pteridine level.

Variable	Overall subjects	Pteridines		
		1st tertile	2nd tertile	3rd tertile
Number	258	86	86	86
Age (years)	45.8 ± 8.1	44.0 ± 7.2	45.8 ± 8.3	47.6 ± 8.3*
Smokers (%)	43.4	30.2	34.9	65.1**^,††^
Drinkers (%)	66.3	68.6	65.1	65.1
Regular exercise (%)	18.6	22.1	17.4	16.3
Therapy for hypertension (%)	9.7	12.8	11.6	4.7
Therapy for dyslipidemia (%)	4.7	4.7	9.3	0^††^
Therapy for diabetes mellitus (%)	2.3	1.2	2.3	3.5
Body mass index (kg/m^2^)	23.2 ± 3.1	23.1 ± 3.2	23.6 ± 3.0	23.0 ± 2.9
Systolic blood pressure (mmHg)	117.5 ± 16.3	120.0 ± 18.8	117.7 ± 15.4	114.8 ± 14.1
Diastolic blood pressure (mmHg)	75.4 ± 11.0	76.2 ± 12.2	75.2 ± 10.6	75.0 ± 10.3
Fasting blood sugar (mg/dl)	96.3 ± 14.2	95.8 ± 10.5	95.1 ± 12.1	98.2 ± 18.6
Triglycerides (mg/dl)	84.5 (62.0, 126.0)	92.5 (63.8, 130.8)	86.5 (60.8, 125.3)	78.5 (62.8, 120.0)
HDL cholesterol (mg/dl)	59.3 ± 13.1	58.4 ± 12.2	60.1 ± 12.7	59.4 ± 14.2
LDL cholesterol (mg/dl)	117.6 ± 29.7	117.5 ± 29.8	119.2 ± 27.3	116.2 ± 32.0
eGFR (ml/min/1.73 m^2^)	74.1 ± 12.6	72.5 ± 12.4	74.5 ± 14.5	75.3 ± 10.8
DNA/RNA oxidation products (μM/g creatinine)	87.1 (71.3, 117.4)	78.0 (60.0, 101.5)	83.0 (71.4, 113.6)	107.0 (83.9, 133.5)**^,††^
15-isoprostane F_2t_ (μM/g creatinine)	1.47 (0.98, 2.22)	1.25 (0.68, 1.64)	1.67 (1.01, 2.40)**	1.76 (1.22, 3.02)**
Urinary pteridines (μM/g creatinine)	14.3 (11.0, 19.3)	10.2 (8.8, 11.0)	14.3 (13.2, 15.2)**	22.9 (19.2, 27.0)**^,††^

Numbers, percentages, means with standard deviations, and medians with 25 and 75 percentile values are shown.

Symbols denote significant differences from the 1st tertile group (*p < 0.05; **p < 0.01) and the 2nd tertile group (^††^*p* < 0.01) for pteridines.

### Comparisons of urinary levels of DNA/RNA oxidation products and 15-isoprostane F_2t_ among the three tertile groups for pteridines

Table 1 also shows comparisons of urinary levels of DNA/RNA oxidation products and 15-isoprostane F_2t_ among the three tertile groups for pteridines in univariate analysis. The median urinary level of DNA/RNA oxidation products was significantly higher in the 3rd tertile group for pteridines than in the 1st and 2nd tertile groups. The median urinary level of 15-isoprostane F_2t_ was significantly higher in the 2nd and 3rd tertile groups than in the 1st tertile group. Similar results were obtained in the multivariate analysis for comparisons of DNA/RNA oxidation products and 15-isoprostane F_2t_ among the pteridine tertile groups (Table 2).

**Table 2:** Comparisons of urinary levels of DNA/RNA oxidation products and 15-isoprostane F_2t_ among the tertile groups for urinary pteridines in multivariate analysis.

Variable	Pteridines		
	1st tertile	2nd tertile	3rd tertile
Log(DNA/RNA oxidation products)	1.911 (1.877 to 1.945)	1.934 (1.901–1.967)	2.015 (1.981–2.050)**^,††^
Log(15-isoprostane F_2t_)	0.053 (− 0.008 to 0.114)	0.189 (0.130–0.249)**	0.227 (0.164–0.289)**

Means with their 95% confidence intervals in parentheses are shown. Age, history of smoking, BMI and eGFR were used as other explanatory variables in multivariate analysis.

Symbols denote significant differences from the 1st tertile (**p < 0.01) and 2nd tertile (^††^*p* < 0.01) for pteridines.

### Correlations of urinary pteridine levels with urinary levels of DNA/RNA oxidation products and 15-isoprostane F_2t_

**Figure 3:**
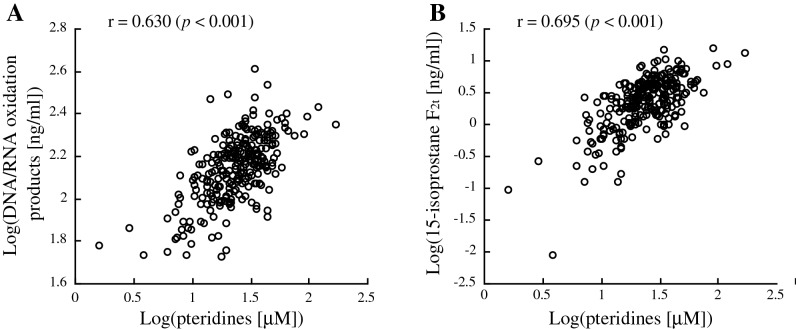
Scatter plots for correlations of log-transformed urinary pteridines with log-transformed urinary DNA/RNA oxidation products (**A**) and 15-isoprostane F_2t_ (**B**). Pearson’s correlation coefficients are shown in the figures.

As shown in Fig. 3, in univariate analysis, log-transformed urinary pteridine levels were significantly correlated with log-transformed urinary levels of DNA/RNA oxidation products and 15-isoprostane F_2t_ (Pearson’s correlation coefficients: 0.630 [*p* < 0.001] for DNA/RNA oxidation products and 0.695 [*p* < 0.001] for 15-isoprostane F_2t_).

Table 3 shows correlations of log-transformed urinary pteridine levels with log-transformed levels of DNA/RNA oxidation products and 15-isoprostane F_2t_ in multiple regression analysis using age, history of smoking, BMI and estimated glomerular filtration rate (eGFR) as other explanatory variables. Log-transformed pteridine levels were significantly correlated with log-transformed levels of DNA/RNA oxidation products and 15-isoprostane F_2t_ (standardized partial regression coefficient: DNA/RNA oxidation products, 0.655 [*p* < 0.001]; 15-isoprostane F_2t_, 0.682 [*p* < 0.001]).

**Table 3:** Correlations of urinary pteridine levels with urinary DNA/RNA oxidation products and 15-isoprostane F_2t_ levels in multiple linear regression analysis.

Variable	DNA/RNA oxidation products	15-isoprostane F_2t_
Pteridines	0.655 (*p* < 0.001)	0.682 (*p* < 0.001)
Age	0.004 (*p* = 0.943)	0.018 (*p* = 0.688)
Smoking	− 0.076 (*p* = 0.143)	0.005 (*p* = 0.914)
Body mass index	− 0.021 (*p* = 0.667)	0.065 (*p* = 0.142)
eGFR	− 0.028 (*p* = 0.584)	0.212 (*p* < 0.001)

Standardized partial regression coefficients of each variable with DNA/RNA oxidation products and 15-isoprostane F_2t_ are shown. Urinary levels of pteridines, DNA/RNA oxidation products and 15-isoprostane F_2t_ were used after logarithmic transformation.

*p* values are given in parentheses.

### Relationships of smoking with urinary levels of pteridines, DNA/RNA oxidation products and 15-isoprostane F_2t_

Table 4 shows comparisons of urinary levels of pteridines, DNA/RNA oxidation products and 15-isoprostane F_2t_ among non-, light and heavy smokers in univariate and multivariate analyses. In univariate analysis, the median values of pteridines and 15-isoprostane F_2t_ were significantly higher in light and heavy smokers than in nonsmokers. The median value of pteridines was also significantly higher in heavy smokers than in light smokers. In multivariate analysis with adjustment for age, BMI and eGFR (ANCOVA), log-transformed values of urinary pteridines, DNA/RNA oxidation products and 15-isoprostane F_2t_ levels were used for comparison. The mean of log-transformed pteridine levels was significantly higher in heavy smokers than in non- and light smokers and was significantly higher in light smokers than in nonsmokers. The mean of log-transformed 15-isoprostane F_2t_ was significantly higher in light and heavy smokers than in nonsmokers. In both univariate and multivariate analyses, there were no significant differences in DNA/RNA oxidation products among non-, light and heavy smokers.

**Table 4:** Comparison of urinary levels of pteridines, DNA/RNA oxidation products and 15-isoprostane F_2t_ among non-, light and heavy smokers.

Variable	Nonsmokers	Light smokers (1–15 cigarettes/day)	Heavy smokers (16 cigarettes or more/day)
**Univariate analysis (μM/g creatinine)**			
Pteridines	13.0 (10.3, 16.2)	15.1 (11.7, 19.6)*	19.2 (14.2, 25.0)**^,†^
DNA/RNA oxidation products	88.4 (68.1, 116.3)	82.9 (71.9, 106.5)	88.4 (64.0, 128.8)
15-isoprostane F_2t_	1.27 (0.79, 1.92)	1.84 (1.20, 2.92)**	1.86 (1.23–2.62)**
**Multivariate analysis**			
Log(pteridines)	1.120 (1.092–1.149)	1.192 (1.142–1.243)*	1.276 (1.232–1.319)**^,†^
Log(DNA/RNA oxidation products)	1.954 (1.928–1.980)	1.947 (1.900–1.993)	1.956 (1.917–1.995)
Log(15-isoprostane F_2t_)	0.086 (0.040–0.133)	0.271 (0.188–0.353)**	0.232 (0.163–0.301)**

For results of univariate analysis, medians with 25 and 75 percentile values in parentheses are shown. For results of multivariate analysis (ANCOVA), means with their 95% confidence intervals in parentheses are shown. Age, BMI and eGFR were used as other explanatory variables in multivariate analysis.

Symbols denote significant differences from nonsmokers (*p < 0.05; **p < 0.01) and light smokers (^†^*p* < 0.05).

### Correlations of urinary level of each pteridine derivative measured by HPLC with urinary levels of fluorometric pteridines, DNA/RNA oxidation products and 15-isoprostane F_2t_

Table 5 shows Pearson’s correlation coefficients in univariate analysis and standardized partial regression coefficients in multivariate analysis between log-transformed levels of each pteridine derivative measured by HPLC and log-transformed levels of fluorometric pteridines, DNA/RNA oxidation products and 15-isoprostane F_2t_. In multivariate analysis, age, history of smoking, BMI and eGFR were used as other explanatory variables. In both univariate and multivariate analyses, log-transformed levels of pteridines, DNA/RNA oxidation products and 15-isoprostane F_2t_ were significantly correlated with log-transformed levels of pterin-6-carboxylic acid, neopterin and biopterin. Log-transformed level of pteridines also showed significant correlations with log-transformed levels of xanthopterin and pterin but not with log-transformed level of isoxanthopterin. Log-transformed levels of DNA/RNA oxidation products and 15-isoprostane F_2t_ also showed significant correlations with log-transformed level of pterin but not with log-transformed level of xanthopterin or isoxanthopterin. Among the six pteridine derivatives measured by HPLC, levels of neopterin and biopterin showed relatively strong correlations with levels of fluorometric pteridines, DNA/RNA oxidation products and 15-isoprostane F_2t_.

**Table 5:** Correlations of each urinary pteridine derivative with urinary pteridines, DNA/RNA oxidation products and 15-isoprostane F_2t_.

Variable	Pteridines	DNA/RNA oxidation products	15-Isoprostane F_2t_
Pterin-6-carboxylic acid	0.509**	0.408**	0.264**
	0.548**	0.413**	0.310**
Neopterin	0.654**	0.531**	0.413**
	0.663**	0.521**	0.437**
Xanthopterin	0.276**	0.112	0.078
	0.269**	0.105	0.035
Isoxanthopterin	0.031	0.174	− 0.104
	0.017	0.161	− 0.133
Biopterin	0.712**	0.505**	0.505**
	0.688**	0.476**	0.511**
Pterin	0.278*	0.401**	0.199*
	0.309**	0.396**	0.204*

Shown are Pearson’s correlation coefficients in univariate analysis (upper lines) and standardized partial regression coefficients in multivariate analysis (lower lines) between each pair of the variables. In multivariate analysis, age, history of smoking, BMI and eGFR were used as other explanatory variables.

Asterisks denote significant correlations (*p < 0.05; **p < 0.01).

### Correlations of urinary pteridines and oxidative stress biomarkers with other cardiovascular risk factors

Correlations of each pteridine derivative measured by HPLC with other cardiovascular risk factors, including BMI, blood pressure (systolic and diastolic), fasting blood sugar, triglycerides, HDL cholesterol and LDL cholesterol, were investigated by linear regression analysis. As shown in Supplementary Table S1, there were no significant correlations between all of the pairs of variables except for a weak correlation between LDL cholesterol and xanthopterin (*r* = 0.248 [*p* = 0.013]). Correlations of the three urinary biomarkers (spectrofluorometric pteridines, DNA/RNA oxidation products and 15-isoprostane F_2t_) with the above cardiovascular risk factors were also investigated. As shown in Supplementary Table S2, there were no significant correlations between all of the pairs of variables.

## Discussion

Using a simple method of spectrofluorometry, we estimated pteridine level in urine and, for the first time, demonstrated that the fluorometric pteridine level is associated with levels of known oxidative stress markers, DNA/RNA oxidation products and 15-isoprostane F_2t_, in healthy middle-aged men. The purpose of this study was to estimate the degree of oxidative stress in vivo by measuring naturally oxidized forms of pteridines in urine. Thus, we needed to measure only oxidized-form pteridines in urine. It is convenient that the oxidized forms of pteridine represented by neopterin, but not its reduced form, emits fluorescence^10^. Thus, urine samples were used for estimation of pteridines by spectrofluorometry without oxidization pretreatment. The correlations of pteridines with DNA/RNA oxidation products and 15-isoprostane F_2t_ were considerably strong with standardized partial correlation coefficients being 0.655 and 0.682, respectively, and were not confounded by age, smoking, BMI and eGFR (Table 3). In addition, the fluorometric pteridine level was higher in smokers than in nonsmokers (Table 4), and the percentage of smokers was higher in the highest tertile group for pteridines than in the lower tertile groups (Table 1). These findings agree with the fact that smoking increases oxidative stress in vivo^16,17^. Accordingly, we here propose urinary spectrofluorometrical pteridine level as a useful biomarker for oxidative stress. Because of its convenience and cost-effectiveness, fluorometric measurement of urinary oxidized pteridines is thought to be suitable for a screening test to evaluate individual oxidative stress level. In the present study, there were no associations between urinary pteridines and cardiovascular risk factors. Similarly, no associations were found between the known oxidative stress markers (DNA/RNA oxidation products and 15-isoprostane F_2t_) and cardiovascular risk factors. These results indirectly support our hypothesis that urinary pteridine level is a biomarker reflecting oxidative stress in vivo.

Blood folate level is known to be lower in smokers than in nonsmokers^21,22^. However, it remains unknown whether and how urinary pteridines are influenced by smoking. In the present study, fluorometric pteridine levels in urine were clearly shown to be higher in smokers than in nonsmokers. As shown in Table 4, a dose–response relationship was shown between smoking and pteridines but not between smoking and 15-isoprostane F_2t_, and there was no association between smoking and DNA/RNA oxidation products. Thus, when fluorometric pteridines and the oxidative stress markers tested in this study were compared regarding their relations with smoking, the relation of pteridines was stronger than the relations of DNA/RNA oxidation products and 15-isoprostane F_2t_. Therefore, the urinary level of fluorometric pteridines is thought to reflect oxidative stress in vivo more strongly than do DNA/RNA oxidation products and 15-isoprostane F_2t_. Although 8-hydroxy-2′-deoxyguanosine as well as 15-isoprostane F_2t_ was reportedly higher in smokers than in nonsmokers^23,24^, there was no association between smoking and DNA/RNA oxidation products in the present study. The reason for this disagreement is unknown, but one possible explanation is a low percentage of participants who were heavy smokers in this study: the proportion of very heavy smokers consuming 30 or more cigarettes per day was only 3.1% (n = 8).

Several methods have been used for measurement of pteridines including HPLC, gas chromatography and mass spectrometry^7^. Trehan and Noronha developed a simple method by fluorometry for measurement of urinary pteridines using wavelengths of 450 mm for emission and 360 nm for excitation^20^. In their original method to estimate total preridine level, reduced-form pteridine in a urine sample was oxidized with iodine before measurement since the oxidized form of pteridine, but not its reduced form, emits fluorescence^10^. Since our purpose was to measure only oxidized-form pteridines in urine reflecting oxidative stress in vivo, oxidization treatment of urine samples was not needed in the present study, and the above wavelengths used in the original method were confirmed to be optimum also for measurement of oxidized pteridines in urine (Fig. 1). According to the results of HPLC analysis, the main pteridine derivatives reflected by fluorometric pteridine level in this study were neopterin and biopterin. This agrees with the results of correlations between each pteridine derivative and fluorometric pteridines: Neopterin and biopterin showed relatively strong correlations with fluorometric pteridines (Table 5). Interestingly, neopterin and biopterin also showed relatively strong correlations with DNA/RNA oxidation products and 15-isoprostane F_2t_. Therefore, these pteridine derivarives are thought to reflect oxidative stress more strongly than do other derivatives such as xanthopterin, isoxanthopterin and pterin, suggesting that our spectrofluorometric analysis for oxidized-form pteridines is highly efficient for detection of oxidative stress in vivo.

There are limitations of this study: The number (n = 258) and age range (25–65 years) of the subjects in this study are limited, and further studies with subjects from a larger cohort and of different ages are therefore needed to confirm the findings of this study. The participants of this study were healthy Japanese men. Thus, further studies in women and ethnically and/or racially different persons are also needed to confirm the findings. Moreover, it would be interesting to investigate the relationship between pteridine level and oxidative stress under pathological conditions, and further studies are needed to determine the cut-off value of urinary pteridines in a larger population. We evaluated the current status of smoking, but no information on ex-smokers was available. However, it is speculated that the effect of smoking on oxidative stress in vivo is rather transient and that a past history of smoking does not greatly affect current oxidative stress level. In fact, in a previous study, similar results for the relations of smoking with urinary levels of oxidative stress biomarkers were obtained when ex-smokers were excluded from the non-smoker group^24^. The main species affecting fluorescent intensity around the excitation and emission wavelengths of 360 and 450 nm, respectively, have been reported to be pteridine derivatives (biopterin and neopterin) and kynurenine derivatives (kynurenine, 5-hydroxykynurenine and 3-hydroxykynurenine)^25^. In our preliminary experiments, the fluorescence intensities of kynurenine at an excitation wavelength of 360 nm and emission wavelengths from 400 to 540 nm were much lower (less than 1%) than those of neopterin and biopterin. Moreover, a transient oxidative burst induced by a short-term exhaustion exercise has recently been reported to result in a significant increase in neopterin, but not kynurenine, in blood^26^. Therefore, kynurenine may not contribute to oxidative stress level estimated by fluorometrical measurement in the present study. Future studies are needed to determine whether kynurenine derivatives in urine are related to reactive oxygen species and interfere with the association between urinary pteridine derivatives and oxidative stress. Although the main species affecting fluorescent intensity around the above wavelengths have been reported to be pteridine derivatives^25^, other potential fluorescent compounds may affect the measurement at the wavelengths (excitation, 360 nm; emission, 450 nm) we used for pteridine measurement. The ratio of pteridine compounds in urine (neopterin/biopterin ratio) was shown to be strongly affected by different oxidation procedures^27^. It would thus be interesting in future study to test the significance of the ratio of an oxidized form of pteridine and a reduced form of pteridine in evaluation of oxidative stress generation in vivo. Finally, the present study is cross-sectional in its design and future prospective studies are needed to discuss the causal relationship between pteridines and oxidative stress.

## Conclusion

In this pilot study, the urinary fluorescence level mainly including fluorescent intensities caused by pteridines was shown to be potently associated with known oxidative stress markers and smoking. We propose spectrofluorometrical estimation of urinary pteridines as a simple and useful method for evaluation of oxidative stress in vivo.

## Methods

### Participants

The participants were 258 healthy middle-aged men (mean age: 45.8 ± 8.1 years) who were receiving annual health-checkup examinations at their workplace of a printing company. None of the subjects were engaged in factory work using organic materials or other harmful chemicals, and none of the subjects had a history of cardiovascular or other serious diseases. The protocol of this study was approved by the Ethics Committee of Hyogo College of Medicine. This study was conducted according to the principles of the Declaration of Helsinki. All methods were performed in accordance with the relevant guidelines and regulations. Informed consent was obtained from all subjects participating in the study.

Histories of cigarette smoking, alcohol drinking, physical activity, illness, and medication therapy were surveyed by questionnaires. Persons receiving therapy for any diseases were excluded from the subjects of this study. In smokers, current smoking status was classified into light smokers (less than 16 cigarettes consumed per day) and heavy smokers (16 cigarettes or more consumed per day). Alcohol drinking was evaluated by frequency of drinking, which was classified into never, occasional and regular drinkers. Physical activity was evaluated by frequency of habitual exercise (30 min per day or longer), which was classified into never, 1–3 times per month, 1–2 times per week, and 3 or more times per week. Blood and urine samples were collected in the morning on the day of the health checkup after overnight fasting and stored at − 80 degrees until measurements.

### Measurements of oxidative stress-related substances in urine samples

Levels of oxidized-form pteridine derivatives in urine were estimated by using a spectrofluorometer (Spectra Max Gemini EM; Molecular Devices, Sunnyvale, CA, USA) with an excitation wavelength of 360 nm and an emission wavelength of 450 nm. No pretreatment including oxidization of urine samples was performed. Urine sample was diluted to a ratio of 1:100 with 10 mM HEPES buffer (pH 7.0) before spectrofluorometry. A standard curve for measurement of pteridines was prepared using neopterin as a standard substance at concentrations of 0, 6.25, 12.5, 25, 50 and 100 μM. In our preliminary study, the inner filter effect was checked by measuring levels of neopterin used as a standard in differently diluted samples of urine (n = 8). As shown in Supplementary Fig. S1, there was an almost linear relationship between urine dilution (1/10–1/100) and neopterin levels in urine. Similar results were obtained when other pteridine derivatives were used as standards. Therefore, the inner filter effect is negligible in our system (100-fold dilution) of urinary pteridine measurement. Moreover, in a previous study, 50-fold dilution has been shown to be suitable for spectrofluorometric measurement of pteridines^20^. Thus, 100-fold dilution of urine samples is enough for avoiding inner filter effect in spectrofluorometry for urinary pteridines.

Levels of DNA/RNA oxidation damage products and 15-isoprostane F_2t_ were measured by enzyme-linked immunoassays using commercial kits, DNA/RNA Oxidation Damage (High Sensitivity) ELISA kit (Cayman Chemical, Ann Arbor, MI, USA) and Urinary Isoprostane EIA Kit (Oxford Biochemical Research Inc., Oxford, MI, USA), respectively.

### Analysis of pteridine derivatives in urine by high-performance liquid chromatography (HPLC)

Urinary levels of major pteridine derivatives, including pterin-6-carboxylic acid, neopterin, biopterin, xanthopterin, isoxanthopterin and pterin, were measured by HPLC using an alliance HPLC system with a 2475 fluorescence detector and Empower chromatography data software (Waters Corporation, Milford MA, USA). The temperature of the column (COSMOSIL 5C18 packed column, 4.6 × 250 mm, Nacalai Tesque, Kyoto, Japan) was kept at 40 degrees. Each urine sample was diluted to a ratio of 1:10 with distilled water and filtrated with a 0.22 μm filter, and then an aliquot (5 μl) of the sample was injected into the HPLC system. Elution was performed at a flow rate of 0.8 ml/min using a solution consisting of phosphate buffer (10 mM, pH 6.5) and methanol at a ratio of 92 (phosphate buffer): 8 (methanol). Pteridines in an eluent were detected using fluorescence at excitation and emission wavelengths of 380 nm and 460 nm, respectively, and were quantified by using commercial standards of pterin-6-carboxylic acid (Sigma-Aldrich Japan, Tokyo, Japan), neopterin (Cayman Chemical), biopterin (Cayman Chemical), isoxanthopterin (Sigma-Aldrich Japan), xanthopterin (Tokyo Chemical Industry Co., Ltd. Tokyo, Japan) and pterin (Sigma-Aldrich Japan).

### Measurements of other variables

Height and body weight were measured with the subjects wearing light clothes at the health checkup. Body mass index (BMI) was calculated as weight in kilograms divided by the square of height in meters. Systolic blood pressure and diastolic blood pressure were measured in a sitting position after at least 5 min of rest.

LDL cholesterol, HDL cholesterol, triglycerides, blood sugar, and creatinine concentrations in serum were automatically measured by conventional methods. In order to evaluate renal function, estimated glomerular filtration rate (eGFR) was calculated by using the following equation developed by the Japanese Society of Nephrology: eGFR (ml/min/1.73 m^2^) = 194 × serum creatinine^−1.094^ × age^−0.287^^28^.

### Statistical analysis

Results are shown as means with standard deviations, means with 95% confidence intervals, or medians with interquartile ranges. The values of urinary fluorometric pteridines in overall participants were arranged in ascending order, and then the participants were divided into three tertile groups of equal sizes. Each variable was compared among the tertile groups for pteridines. Categorical variables were compared between each pair of the tertiles for pteridines using the chi-square test for independence. In univariate analysis, comparisons of means of each variable in the tertile groups for urinary pteridine levels and in the different smoking groups were performed by analysis of variance and subsequent Scheffé's F-test. In multivariate analysis, mean values were compared using analysis of covariance (ANCOVA) and subsequent Student's t-test after Bonferroni correction. Since the levels of triglycerides, urinary pteridines, DNA/RNA oxidation products, 15-isoprostane F_2t_ and each pteridine derivative did not show a normal distribution, they were compared non-parametrically by using the Kruskal–Wallis test followed by the Steel–Dwass test in univariate analysis or were used after logarithmic transformation in ANCOVA. Pearson's correlation coefficients and standardized partial correlation coefficients were calculated in simple and multiple linear regression analyses, respectively. The values of triglycerides, urinary pteridines, DNA/RNA damage products, 15-isoprostane F_2t_ and each pteridine derivative were also used after logarithmic transformation in linear regression analysis. Probability (*p*) values less than 0.05 were defined as significant.

## Supplementary information

Supplementary Tables

Supplementary Figure 1
