# Supplementary material for: A simple and useful method for evaluation of oxidative stress in vivo by spectrofluorometric estimation of urinary pteridines
Source: Sci Rep. 2020 Jul 8;10:11223. doi: 10.1038/s41598-020-67681-4 (PMC7343776; doi:10.1038/s41598-020-67681-4)
Supplement: Supplementary file 1 — Supplementary Tables [file 41598_2020_67681_MOESM1_ESM.docx]

**Supplementary Tables 1 and 2**

**Title:** A simple and useful method for evaluation of oxidative stress in vivo by spectrofluorometric estimation of urinary pteridines

**Authors:** Ichiro Wakabayashi^1^, Mamoru Nakanishi^2^, Makoto Ohki^2^, Akira Suehiro^3^, Kagehiro Uchida^2^

**From:**

^1^Department of Environmental and Preventive Medicine, Hyogo College of Medicine, Hyogo, 663-8501, Japan.

^2^Mibyoumarker Laboratory Co., Ltd., Osaka, 530-0043, Japan.

^3^General Education Center, Hyogo University of Health Science, Hyogo 650-8530, Japan.

**Corresponding author:**

Ichiro Wakabayashi, MD & PhD

Department of Environmental and Preventive Medicine,

Hyogo College of Medicine,

Mukogawa-cho 1-1, Nishinomiya, Hyogo 663-8501, Japan

Tel: +81-798-45-6561; Fax: +81-798-45-6563

E-mail: wakabaya@hyo-med.ac.jp

**Supplementary Table 1.**

Correlations of each pteridine derivative with other biomarkers related to cardiovascular disease.

|  | Pterin-6-carboxylic acid | Neopterin | Xanthopterin | Isoxantopterin | Biopterin | Pterin |
| --- | --- | --- | --- | --- | --- | --- |
| BMI | 0.113 | -0.086 | 0.073 | 0.053 | -0.189 | 0.027 |
| SBP | -0.158 | -0.018 | 0.035 | 0.088 | -0.076 | 0.125 |
| DBP | -0.012 | -0.013 | 0.067 | 0.039 | -0.043 | 0.005 |
| Fasting BS | -0.075 | 0.092 | 0.183 | 0.011 | -0.036 | -0.042 |
| Triglycerides | -0.080 | 0.027 | 0.107 | 0.127 | -0.053 | 0.113 |
| HDL-C | -0.151 | -0.142 | 0.029 | -0.018 | 0.017 | 0.088 |
| LDL-C | 0.051 | -0.010 | 0.248* | 0.122 | -0.068 | -0.016 |

Shown are Pearson’s correlation coefficients between each pteridine derivative level and other biomarker levels. Each pteridine level (μM/g creatinine) measured by HPLC and triglyceride level were used after log-transformation since their levels did not show normal distribution. BMI, body mass index; SBP, systolic blood pressure; DBP, diastolic blood pressure; BS, blood sugar; HDL-C, HDL cholesterol; LDL-C, LDL cholesterol. Asterisks denote significant correlations (*, *p* < 0.05).

**Supplementary Table 2.**

Correlations of urinary pteridines, DNA/RNA oxidation products and 15-isoprostane F_2t_ with other biomarkers related to cardiovascular disease.

|  | Pteridines | DNA/RNA oxidation products | 15-isoprostane F_2t_ |
| --- | --- | --- | --- |
| BMI | -0.038 | 0.030 | 0.027 |
| SBP | -0.117 | 0.096 | -0.108 |
| DBP | -0.018 | 0.133 | -0.070 |
| Fasting BS | 0.081 | 0.107 | 0.025 |
| Triglycerides | -0.023 | 0.062 | -0.067 |
| HDL-C | 0.040 | 0.051 | 0.022 |
| LDL-C | -0.022 | 0.037 | -0.106 |

Shown are Pearson’s correlation coefficients between each pteridine derivative level and other biomarker levels. Levels of pteridines, DNA/RNA oxidation products, 15-isoprostane F_2t_ and triglycerides were used after log-transformation since their levels did not show normal distributions. BMI, body mass index; SBP, systolic blood pressure; DBP, diastolic blood pressure; BS, blood sugar; HDL-C, HDL cholesterol; LDL-C, LDL cholesterol.
