# Supplementary material for: A simple and useful method for evaluation of oxidative stress in vivo by spectrofluorometric estimation of urinary pteridines
Source: Sci Rep. 2020 Jul 8;10:11223. doi: 10.1038/s41598-020-67681-4 (PMC7343776; doi:10.1038/s41598-020-67681-4)
Supplement: Supplementary file 2 — Supplementary Figure 1 [file 41598_2020_67681_MOESM2_ESM.pptx]

## Slide 1
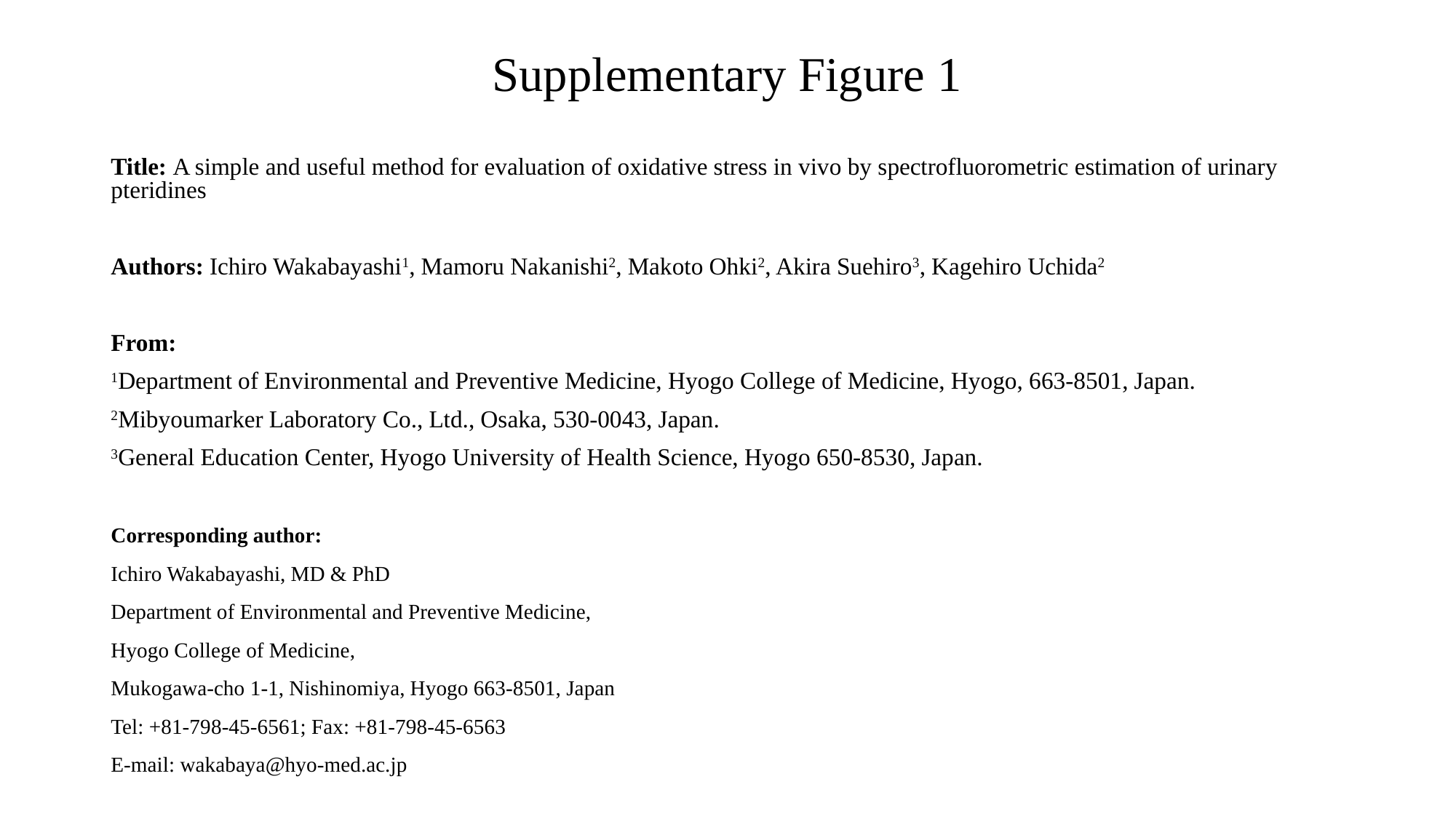

# Supplementary Figure 1
Title: A simple and useful method for evaluation of oxidative stress in vivo by spectrofluorometric estimation of urinary pteridines
Authors: Ichiro Wakabayashi1, Mamoru Nakanishi2, Makoto Ohki2, Akira Suehiro3, Kagehiro Uchida2
From:
1Department of Environmental and Preventive Medicine, Hyogo College of Medicine, Hyogo, 663-8501, Japan.
2Mibyoumarker Laboratory Co., Ltd., Osaka, 530-0043, Japan.
3General Education Center, Hyogo University of Health Science, Hyogo 650-8530, Japan.
Corresponding author:
Ichiro Wakabayashi, MD & PhD
Department of Environmental and Preventive Medicine,
Hyogo College of Medicine,
Mukogawa-cho 1-1, Nishinomiya, Hyogo 663-8501, Japan
Tel: +81-798-45-6561; Fax: +81-798-45-6563
E-mail: wakabaya@hyo-med.ac.jp

## Slide 2
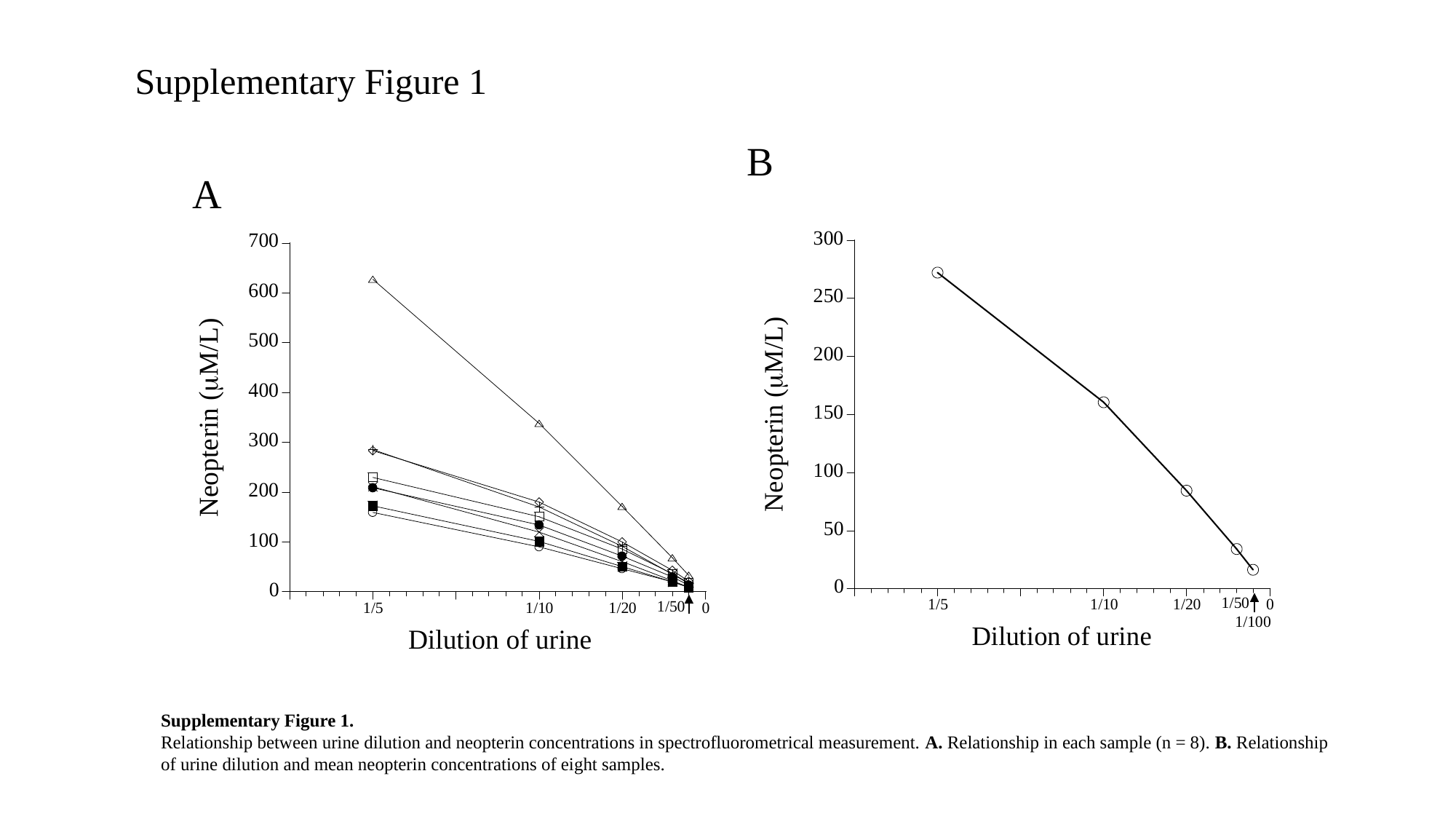

Supplementary Figure 1
Supplementary Figure 1.
Relationship between urine dilution and neopterin concentrations in spectrofluorometrical measurement. A. Relationship in each sample (n = 8). B. Relationship of urine dilution and mean neopterin concentrations of eight samples.
